# Development, Validation and Application of an RP-HPLC Method for the Determination of Reproxalap in Cyclodextrin Inclusion Complexes and an In Situ Ocular Hydrogel

**DOI:** 10.3390/pharmaceutics18091068

**Published:** 2026-08-26

**Authors:** Rumeysa Ceylan, Eren Aytekin, Heybet Kerem Polat, Ozan Kaplan, Mustafa Çelebier, Sibel Bozdağ Pehlivan

**Affiliations:** 1 Department of Pharmaceutical Technology, Faculty of Pharmacy, Hacettepe University, Ankara 06230, Turkey; rumeysa.cirak@afsu.edu.tr (R.C.); erenaytekin@hacettepe.edu.tr (E.A.); 2 Department of Pharmaceutical Biotechnology, Afyonkarahisar Health Sciences University, Afyonkarahisar 03030, Turkey; 3 Turkish Medicines and Medical Devices Agency, Republic of Turkiye Ministry of Health, Ankara 06520, Turkey; kerem_hybt@hotmail.com; 4 Department of Analytical Chemistry, Faculty of Pharmacy, Hacettepe University, Ankara 06100, Turkey; ozankaplan@hacettepe.edu.tr (O.K.); celebier@hacettepe.edu.tr (M.Ç.)

**Keywords:** analytical method validation, dry eye disease, reproxalap, cyclodextrin inclusion complex, in situ gel

## Abstract

**Background/Objectives**: The aim of this study was to develop and validate a simple, accurate, reproducible, and sensitive reverse-phase high-performance liquid chromatography method for the quantification of reproxalap (RP) in cyclodextrin (CD) inclusion complexes and poloxamer 407 hydrogel formulations. **Methods**: Chromatographic separation was achieved using a mobile phase of deionized water and an organic phase (methanol:acetonitrile, 55:45 *v*/*v*) in a 40:60 (*v*/*v*) ratio, at a flow rate of 0.9 mL/min and a run time of 10 min. Validation assessed linearity, specificity, accuracy, and sensitivity over a concentration range of 1–60 μg/mL. Phase-solubility studies were conducted to determine the apparent stability constant (K_1:1_), and inclusion complexes were characterized by Fourier transform infrared spectroscopy and differential scanning calorimetry. **Results**: The method demonstrated specificity, linearity, sensitivity, and accuracy within the tested concentration range of 1–60 μg/mL. The apparent stability constants (K_1:1_), calculated from the phase-solubility plot slopes, were 2666.66 M^−1^ for Sulfobutyl Ether β-Cyclodextrin (SBE-β-CD) and 526.3 M^−1^ for Hydroxypropyl-β-Cyclodextrin (HP-β-CD). The solubility of RP in deionized water (0.25 mM) increased 8.6-fold to 2.15 mM with the HP-β-CD inclusion complex and 22.4-fold to 5.6 mM with the SBE-β-CD inclusion complex. The method was successfully applied to determine RP in inclusion complexes and poloxamer 407 hydrogel formulations, with no interference from formulation excipients. **Conclusions**: The validated RP-HPLC method is suitable for quality control analysis of RP in CD inclusion complexes and poloxamer 407 hydrogel formulations, supporting its application in the future development of these drug delivery systems.

## 1. Introduction

Dry eye disease (DED) is a common multifactorial disorder affecting individuals across all age groups, with risk increasing with age. It arises from disruption of tear film homeostasis and is a chronic condition affecting millions worldwide. Prevalence is estimated at 5–50% across countries and populations, and its chronic, progressive nature represents a substantial healthcare burden [1,2,3].

Inflammation plays a significant role in the pathogenesis of dry eye disease (DED), and interrupting the inflammatory cycle is a key strategy for managing DED symptoms [4]. Among the mediators involved, reactive aldehyde species (RASPs) act upstream to amplify pro-inflammatory signaling cascades. Elevated RASP levels have been observed in various ocular inflammatory diseases, including Sjögren’s syndrome, noninfectious uveitis, allergic conjunctivitis, and DED, where they also correlate with symptom severity. In addition to their role in inflammatory signaling, RASPs interact with phosphatidylethanolamine, a key component of the tear lipidome that helps maintain ocular surface moisture. RASPs are therefore considered a promising therapeutic target in DED [5].

Reproxalap (RP) is a novel RASP inhibitor under development to treat DED. It forms covalent bonds with aldehydes, thereby reducing ocular inflammation [6]. RP is a small, crystalline molecule with poor water solubility but is soluble in organic solvents such as methanol and dimethyl sulfoxide. According to Drug Bank data, the IUPAC name of RP is 2-(3-amino-6-chloro-2-quinolinyl) propan-2-ol, and its molecular weight is 236.7 g/mol.

Cyclodextrins (CDs) are cyclic oligosaccharides composed of six, seven, or eight glucose units linked by α-1,4-glycosidic bonds, referred to as α-, β-, and γ-CD, respectively. They are synthesized enzymatically by CD glucosyltransferases [7]. CDs possess a hydrophobic inner cavity and a hydrophilic outer surface, enabling them to noncovalently encapsulate hydrophobic guest molecules as inclusion complexes. β-Cyclodextrins (β-CDs) are the most widely used type because of their lower toxicity relative to other CDs and their cavity size, which is compatible with various guest molecules [8].

Over the past two decades, CDs have been extensively used as host molecules for a range of pharmaceutical guests. By forming inclusion complexes with hydrophobic drugs, they improve solubility and stability [9,10]. Although β-CDs are widely used in pharmaceutical formulations, they are limited by their hemolytic potential and low water solubility. Consequently, several β-CD derivatives have been developed [11]. The most important of these include hydroxypropyl-β-cyclodextrin (HP-β-CD), randomly methylated β-CD, sulfobutyl ether β-cyclodextrin (SBE-β-CD), and glucosyl-β-cyclodextrin [12].

SBE-β-CD is a polyanionic, amorphous, and nonhygroscopic cyclic oligosaccharide. It is synthesized by substituting the primary or secondary hydroxyl groups of β-CD with sulfobutyl groups. Its aqueous solubility is over 50 times that of β-CD, with improved stability and biocompatibility. These properties make it well suited to drug delivery applications that rely on stable inclusion complexes [13].

Inclusion complex formation enhances the physicochemical properties of drug molecules by increasing solubility and thermal stability. During this process, host and guest molecules associate to form supramolecular inclusion complexes through noncovalent interactions, including hydrogen bonding, van der Waals forces, hydrophobic effects, and π–π interactions [14,15].

Hydrogels are hydrophilic, three-dimensional polymer networks widely used to improve ophthalmic drug delivery. By retaining substantial amounts of water while maintaining structural integrity, they protect active ingredients from enzymatic degradation and hydrophobic interactions and provide a stable, hydrophilic environment for drug encapsulation [16]. Thermosensitive hydrogels exhibit temperature-dependent swelling behavior. Following ocular administration, they solidify at a pre-corneal temperature of approximately 35 °C [17]. This sol–gel transition increases resistance to dilution by tears and slows drug drainage. Poloxamer 407 (P407) is commonly used in ocular drug delivery systems. It is an ABA-type block copolymer comprising approximately 70% polyoxyethylene (A block) and 30% polyoxypropylene (B block). Aqueous solutions of P407 exhibit shear-thinning behavior and can be administered safely without damaging ocular tissues [18]. Hyaluronic acid (HA) is a linear polysaccharide composed of d-glucuronic acid and N-acetyl-glucosamine units linked by β-1,4- and β-1,3-glycosidic bonds. Its distinctive viscoelasticity, bioadhesion, biocompatibility, and receptor recognition properties enable its application in various ophthalmic fields, including the treatment of dry eye [19]. İncorporating a mucoadhesive HA polymer into the formulation is a widely used approach to prolong residence time on the ocular surface and enhance the mechanical strength of gels [20].

High-performance liquid chromatography (HPLC) is widely used for quantifying pharmaceutical compounds in formulations and for evaluating drug stability, dissolution, and biological matrices. Developing an analytical method involves optimizing the chromatographic parameters and then validating it. Validation is performed to confirm that the method is suitable for its intended purpose and produces reliable, reproducible results, evaluated against predefined parameters, including accuracy, precision, linearity, specificity, and stability. All relevant national and international guidelines are followed throughout this process [21]. To the best of our knowledge, no validated HPLC method for quantifying RP has been reported to date. Furthermore, the development and characterization of CD inclusion complexes of RP have not been investigated. RP, a novel drug candidate for the treatment of DED, is a small molecule characterized by low water solubility. To address this limitation, RP–cyclodextrin inclusion complexes were developed. These complexes are subsequently incorporated into in situ gel systems. Although RP is analytically amenable to reversed-phase liquid chromatography, no pharmacopoeial monograph, reference analytical procedure, or validated chromatographic method is currently available for its quantitative determination. In addition, its analysis within cyclodextrin-containing systems and poloxamer 407–hyaluronic acid hydrogel matrices requires sufficient chromatographic selectivity to distinguish RP from formulation components and the release medium. Therefore, the analytical contribution of the present study is the development and validation of a fit-for-purpose RP-HPLC procedure for RP and its application throughout an integrated formulation-development workflow, including phase-solubility studies, cyclodextrin complex evaluation, hydrogel analysis, and in vitro release testing. To the best of our knowledge, no cyclodextrin inclusion complex or solubility data was reported for RP, a recently developed small molecule under patent protection, and no pharmacopoeial monograph or reference method is currently available for this compound. The present study addresses this gap by reporting, for the first time, the FTIR and DSC characterization of RP together with the preparation of its cyclodextrin inclusion complex and the associated solubility data, thereby establishing a foundational dataset for future analytical and formulation research. Therefore, the aim of this study was to develop and validate a new, sensitive chromatographic method for RP and to examine its CD inclusion complexes, thereby addressing existing gaps in the literature [11,22,23].

In this study, the analytical method for RP was validated for the active compound RP in accordance with the ICH guideline Q2(R2). Inclusion complexes were prepared using RP: SBE-β-CD inclusion complex (RP: SBE-β-CD IC) and RP: HP-β-CD inclusion complex (RP: HP-β-CD IC) and subsequently characterized. Thermosensitive P407 hydrogels were then prepared using the selected RP: SBE-β-CD IC (RP: SBE-β-CD IC). The in vitro release profile of the formulation was evaluated using the developed reverse-phase high-performance liquid chromatography (RP-HPLC) method.

## 2. Materials and Methods

### 2.1. Materials

RP was purchased from Adooq Bioscience (Irvine, CA, USA). HP-β-CD and Poloxamer 407 were obtained from Sigma-Aldrich (St. Louis, USA). Hyaluronic acid (HA) was purchased from Aromel Kimya (Istanbul, Turkey), and SBE-β-CD was received as a gift sample. All other chemicals were of analytical grade.

### 2.2. Methods

#### 2.2.1. HPLC Conditions

Chromatographic analysis was performed using an Agilent Technologies 200 (Santa Clara, CA, USA) Series HPLC system equipped with a G1311A quaternary pump, a G1322A degasser, an autosampler, and a diode array detector; peak areas were processed using Agilent ChemStation 1.8 software. Separation was achieved on a Supelco C18 column (Sigma-Aldrich, St. Louis, MO, USA). (250 × 4.6 mm, 5 µm, 100 Å). Isocratic elution was performed using a mobile phase of deionized water and an organic phase (methanol:acetonitrile 55:45 *v*/*v*) in a 40:60 (*v*/*v*) ratio at a flow rate of 0.9 mL/min. UV detection was performed at 254 nm, as reported in the manufacturer’s product data sheet (MedChemExpress (Monmouth Junction, NJ, USA)), with a 30 µL injection volume.

#### 2.2.2. Preparation of Calibration Curves

A stock solution of RP was prepared using the mobile phase as the solvent. Calibration curves were constructed under the chromatographic conditions described above. Linearity was determined by analyzing seven concentration levels (1, 2, 5, 10, 20, 40, and 60 μg/mL), with peak areas plotted against the corresponding concentrations. The standard deviations of the slope and intercept, as well as the regression coefficients, were calculated.

#### 2.2.3. Specificity

Specificity was evaluated to confirm the absence of interference from formulation excipients (analytical placebo). Separate placebo solutions were prepared for P407, SBE-β-CD, and HP-β-CD and analyzed in triplicate.

#### 2.2.4. Accuracy

Accuracy was expressed as the percentage deviation from nominal concentration. Standard solutions at known concentration levels (1, 10, and 60 μg/mL) were prepared in triplicate and injected into the system. Percent recovery and relative standard deviation (RSD%) were calculated for each concentration level.

#### 2.2.5. Precision

Method precision was evaluated in terms of repeatability (intraday precision) and intermediate precision (over three consecutive days). RP standard solutions at concentrations of 1, 10, and 60 μg/mL were analyzed in triplicate. RSD% was calculated for each level.

#### 2.2.6. Sensitivity

LOD and LOQ were estimated using the signal-to-noise (S/N) approach. An RP standard solution at a concentration of 1 µg/mL was analysed under the chromatographic conditions described above. The S/N ratio was determined using Agilent ChemStation software by comparing the RP peak signal with the baseline noise measured within a defined region adjacent to the RP retention time. The mean S/N ratio obtained from the 1 µg/mL standard solution was used in the following equations:

(1) $$LOD=\frac{3\times C}{\left(S/N\right)}$$

(2) $$LOQ=\frac{10\times C}{\left(S/N\right)}$$

where C is the concentration of the low-level RP standard solution and S/N is its experimentally measured signal-to-noise ratio.

#### 2.2.7. Solution Stability

The short-term solution stability of RP was evaluated using a 40 µg/mL RP solution prepared in the mobile phase. Aliquots were transferred into tightly sealed chromatographic vials and maintained at room temperature for 48 h. The solutions were analysed at 0, 24, and 48 h under the validated chromatographic conditions. Each measurement was performed in triplicate. Stability was evaluated by comparing the measured RP concentration at each time point with the initial concentration at 0 h. The results were expressed as mean concentration ± SD, percentage of the initial concentration remaining, and RSD%.

#### 2.2.8. Robustness

The robustness of the developed method was evaluated under varying flow rates, temperatures and wavelengths. Standard solutions of RP at a concentration of 35 μg/mL were prepared and analyzed at flow rates of 0.81 and 0.99 mL/min, temperatures of 20 °C and 30 °C and wavelengths of 253 and 254 nm. The results were then compared using statistical analysis. The nominal chromatographic conditions used throughout the study were as follows: column, Supelco C18 column (250 × 4.6 mm, 5 µm, 100 Å), a mobile phase of deionized water and an organic phase (methanol:acetonitrile, 55:45 *v*/*v*) in a 40:60 (*v*/*v*) ratio flow rate, 0.90 mL/min; column temperature, 25 °C; detection wavelength, 254 nm; injection volume, 30 µL. The robustness study was performed using a 35 µg/mL standard solution. This concentration was selected because it lies within the linearity range established for the method (1–60 µg/mL) and is close to the mid-point of this range, making it representative of the concentrations routinely analyzed under the method’s normal operating conditions.

#### 2.2.9. Phase-Solubility Study

Phase-solubility studies of RP with HP-β-CD and SBE-β-CD in aqueous media were conducted as previously described [24]. Excess RP was added to aqueous solutions of HP-β-CD and SBE-β-CD at varying concentrations (0–10 mM). The mixtures were stirred in light-protected flasks at room temperature at 400 rpm for 5 days.

After equilibrium was reached, the samples were centrifuged and filtered through 0.22 μm cellulose acetate (CA) membrane filters to remove undissolved RP. Phase-solubility profiles were constructed by plotting RP solubility as a function of CD concentration. The apparent stability constant (K_1:1_) was calculated from the phase-solubility diagram using the Higuchi–Connors equation:

$$K_{1:1}=\frac{Slope}{\left[S_{0}\times \left(1-slope\right)\right]},$$

where S_0_ is the intrinsic solubility of RP in the absence of CD [25].

#### 2.2.10. Preparation of Inclusion Complexes

İnclusion complexes (RP: SBE-β-CD IC and RP: HP-β-CD IC) were prepared at a 1:1 molar ratio by accurately weighing equimolar amounts of RP and each CD. HP-β-CD and SBE-β-CD were dissolved in deionized water (15 mL), whereas RP was dissolved in methanol (5 mL). The RP solution was then added dropwise to each CD solution using a syringe. The resulting mixtures were mixed for 24 h at 400 rpm at room temperature. Subsequently, the organic solvent was removed using a rotary evaporator, and the resulting solution was frozen at −20 °C and lyophilized at −80 °C for 72 h. The dried complexes were stored in a desiccator until further use [25].

#### 2.2.11. Fourier Transform Infrared Spectroscopy Assay

Fourier transform infrared (FT-IR) spectra of RP, SBE-β-CD, HP-β-CD, the RP: SBE-β-CD and RP: HP-β-CD inclusion complexes, and their corresponding physical mixtures (RP: SBE-β-CD PM and RP: HP-β-CD PM) were recorded using a PerkinElmer Spectrum BX FT-IR spectrometer over the range of 400–4000 cm^−1^ [26].

#### 2.2.12. Differential Scanning Calorimetry

Thermal analysis of the inclusion complexes and physical mixtures was performed using a differential scanning calorimetry (DSC) Q100 calorimeter. Approximately 5 mg of each sample was sealed in aluminum pans and heated at 10 °C/min under a nitrogen atmosphere over a temperature range of 10–400 °C [25].

#### 2.2.13. Solubility Study

The solubility of RP: SBE-β-CD IC and RP: HP-β-CD IC was evaluated as previously described. Excess amounts of each complex were added to deionized water and shaken at 25 °C for 24 h. The samples were then centrifuged and filtered through 0.45-μm CA filters to remove undissolved RP. RP concentration in the supernatant was quantified using the RP-HPLC method which was developed in this study [27].

#### 2.2.14. Characterization of Inclusion Complexes: Moisture Content and Yield

Moisture content was determined by the loss on drying method. Powder samples on aluminum plates at 105 °C until a constant weight was achieved, with the weight loss used for calculation [28]. The yield of the inclusion complexes was determined by weighing the collected product and calculated using the equation below [29].

$$Yield\;(\%)=\frac{Practical\;Mass\;}{Theoretical\;Mass\;(Drug+Carrier)}\times 100\;$$

#### 2.2.15. Preparation of In Situ Gel Formulation

Thermosensitive and mucoadhesive in situ gel formulations were prepared using the cold method with P407 (18% *w*/*v*) and HA (0.2% *w*/*v*) as the mucoadhesive agent, as previously reported. P407 and HA were dissolved in distilled water and stirred overnight at 4 °C using a magnetic stirrer. The RP: SBE-β-CD IC was subsequently added to the mixture [30].

#### 2.2.16. Characterization of In Situ Gel Formulation

A pH meter (Hanna, Vöhringen, Germany) was used to measure the pH. Three measurements were performed (n = 3). The clarity of the hydrogel formulation after gelation was evaluated by holding the sample up to bright light and comparing it against a dark background. Viscosity measurements of hydrogel formulations were conducted using Brookfield Viscometry (DV2T-RV). The viscosity studies of the hydrogel were conducted at 25, 27, 29 and 33 °C. CP52 was selected as the spindle due to the high viscosity of the samples, and 0.5 mL of the sample was used in accordance with the spindle specifications. Rheological characterization of the hydrogel was performed using a Brookfield DV2T-RV viscometer (Essex, UK) equipped with a CP52 spindle. Viscosity determinations were conducted at the corresponding gelation temperature, with measurements recorded across a range of spindle angular velocities, namely 1, 2.5, 5, 10, 20, 50, and 100 rpm [31,32,33].

#### 2.2.17. In Vitro Release Studies

In vitro release was evaluated using the dialysis membrane method. The experiments were performed in phosphate buffer (pH 7.4) containing 0.2% sodium lauryl sulfate. One milliliter of in situ gel was placed in dialysis bags (molecular weight cutoff: 14,000 Da), which were then immersed in 15 mL Falcon tubes containing sufficient release medium to maintain sink conditions. The experiments were conducted in triplicate in a horizontal shaking water bath at 100 rpm and 34 ± 0.5 °C. Samples were collected at predetermined time points, transferred to vials, and analyzed using the RP-HPLC method described above [34].

#### 2.2.18. In Vitro Release Kinetic Studies

DDSolver 1.0 Software was employed to identify the mathematical models associated with the formulations. Forty-five models of formulation release kinetics were evaluated using data from in vitro release studies. Four parameters—R^2^, adjusted R^2^ (R^2^adj), model selection criterion (MSC), and Akaike information criterion (AIC)—were used to assess model fit. The model demonstrating the highest R^2^, R^2^adj, and MSC values, along with the lowest AIC, was selected as the best-fitting mathematical model for the formulations [35].

## 3. Results and Discussion

**Figure 1:**
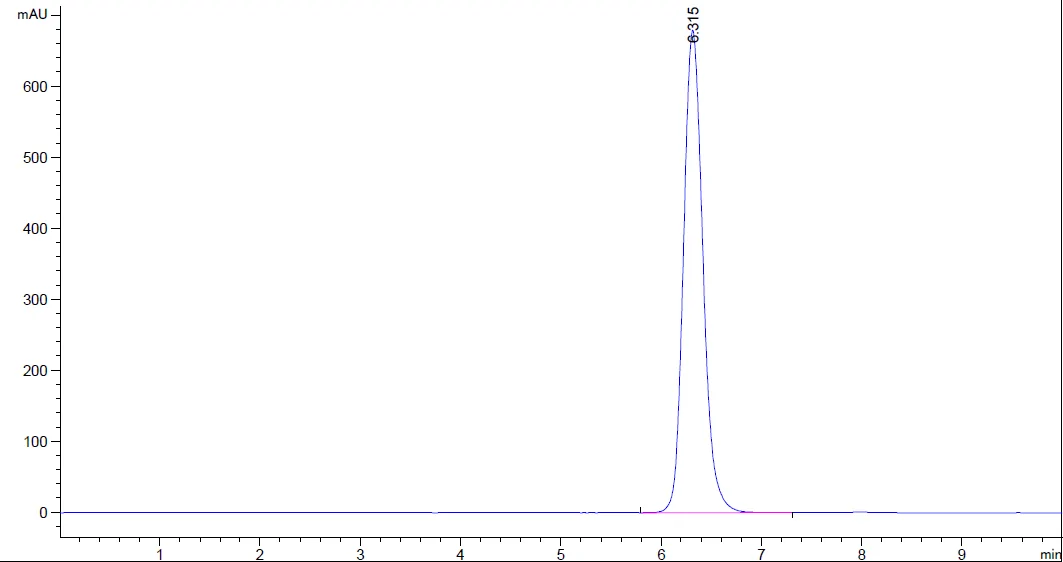
Chromatogram of Reproxalap Standard Solution (40 µg/mL).

No HPLC method has previously been reported for the analysis of RP, either as a pure compound or in formulations. The objective of this study was therefore to develop a reliable method for quantifying RP in P407 hydrogel formulations. According to DrugBank (www.drugbank.com), RP has a log *p* value of 2.22. Given its lipophilicity, a C18 column was selected. During chromatographic method development, the proportion of deionized water to the organic phase was varied while the methanol ratio within the organic phase was maintained at 55:45 (*v*/*v*). At water ratios of 20:80 and 30:70 (*v*/*v*), the retention times of RP were 3.90 and 5.13 min, respectively. Although increasing the organic-phase proportion shortened the retention time, the RP peak eluted closer to the early chromatographic region. The selected 40:60 (*v*/*v*) water ratio produced an RP retention time of 6.45 min and provided an appropriate balance between chromatographic retention, separation from placebo components, reproducible peak integration, and total analysis time (Figure 1). Therefore, this composition was selected for method validation and subsequent sample analysis. Under the experimental conditions, an acceptable response was obtained when the method was selective for RP. Under optimum conditions, the system suitability parameters were as follows: retention time (min) 6.45; capacity factor (k’) 5.72; efficiency (N) 19,126; tailing factor 1.50; and resolution 37.56.

### 3.1. Method Validation

#### 3.1.1. Specificity

**Figure 2:**
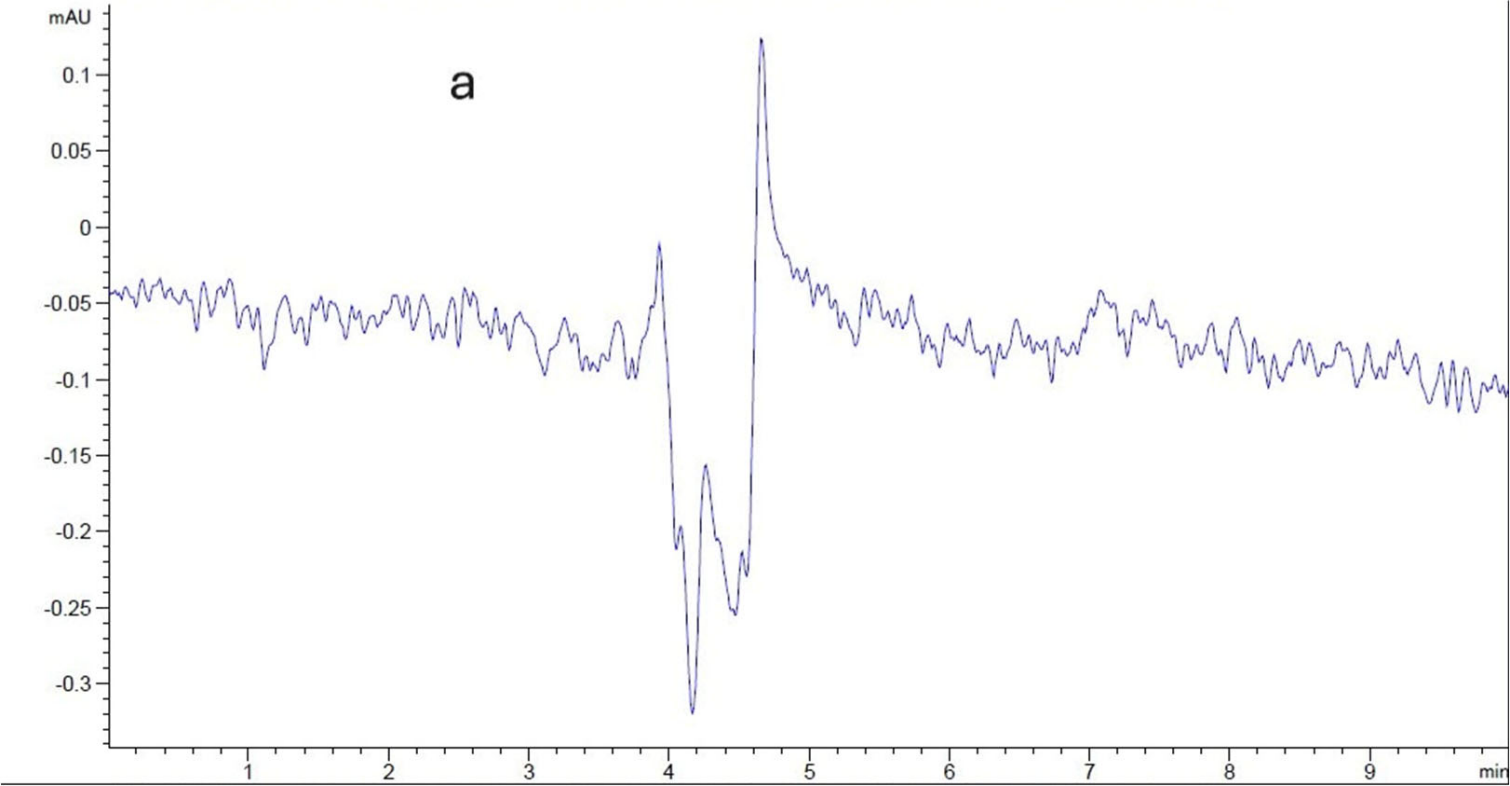

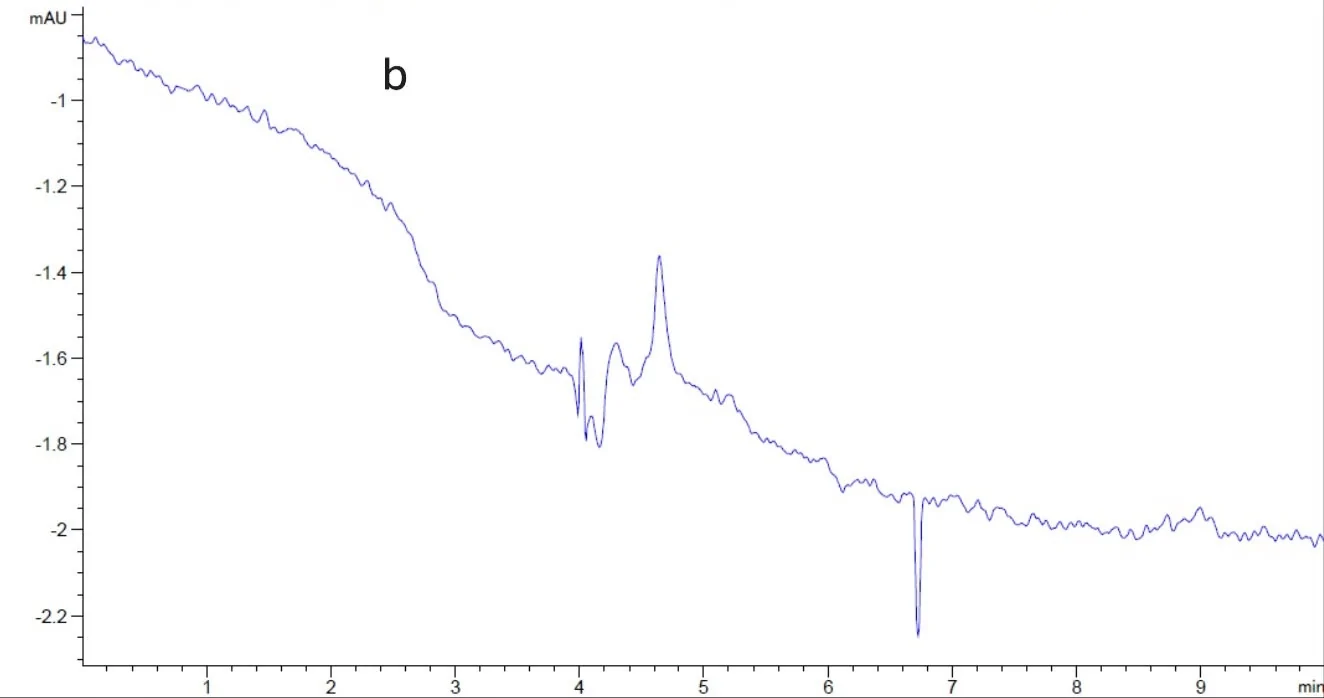

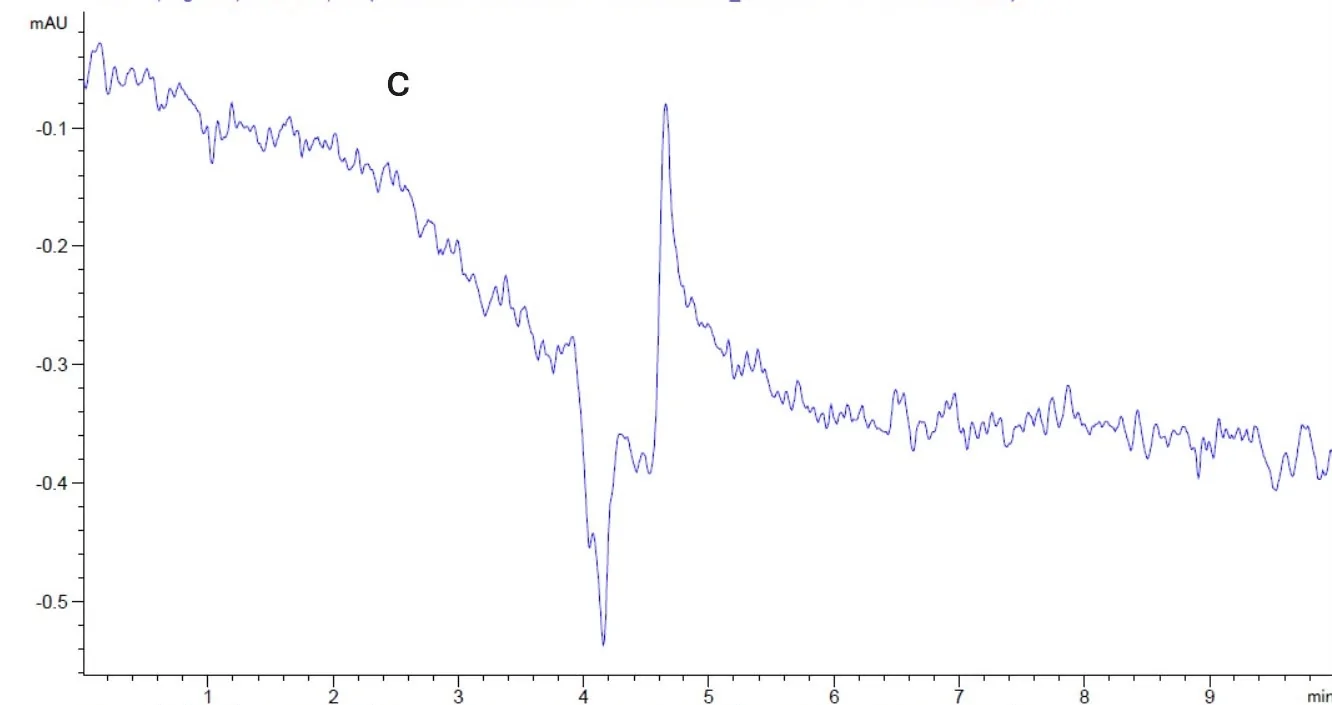
Chromatograms of SBE-β-CD (**a**), HP-β-CD (**b**), and Poloxamer 407 (**c**).

Specificity refers to the ability of an analytical method to accurately quantify the analyte in the presence of other components. In this study, chromatograms of RP and the formulation excipients were evaluated following sequential analysis. No interference from P407, HP-β-CD, or SBE-β-CD was observed at the RP retention time (Figure 2).

#### 3.1.2. Linearity

**Figure 3:**
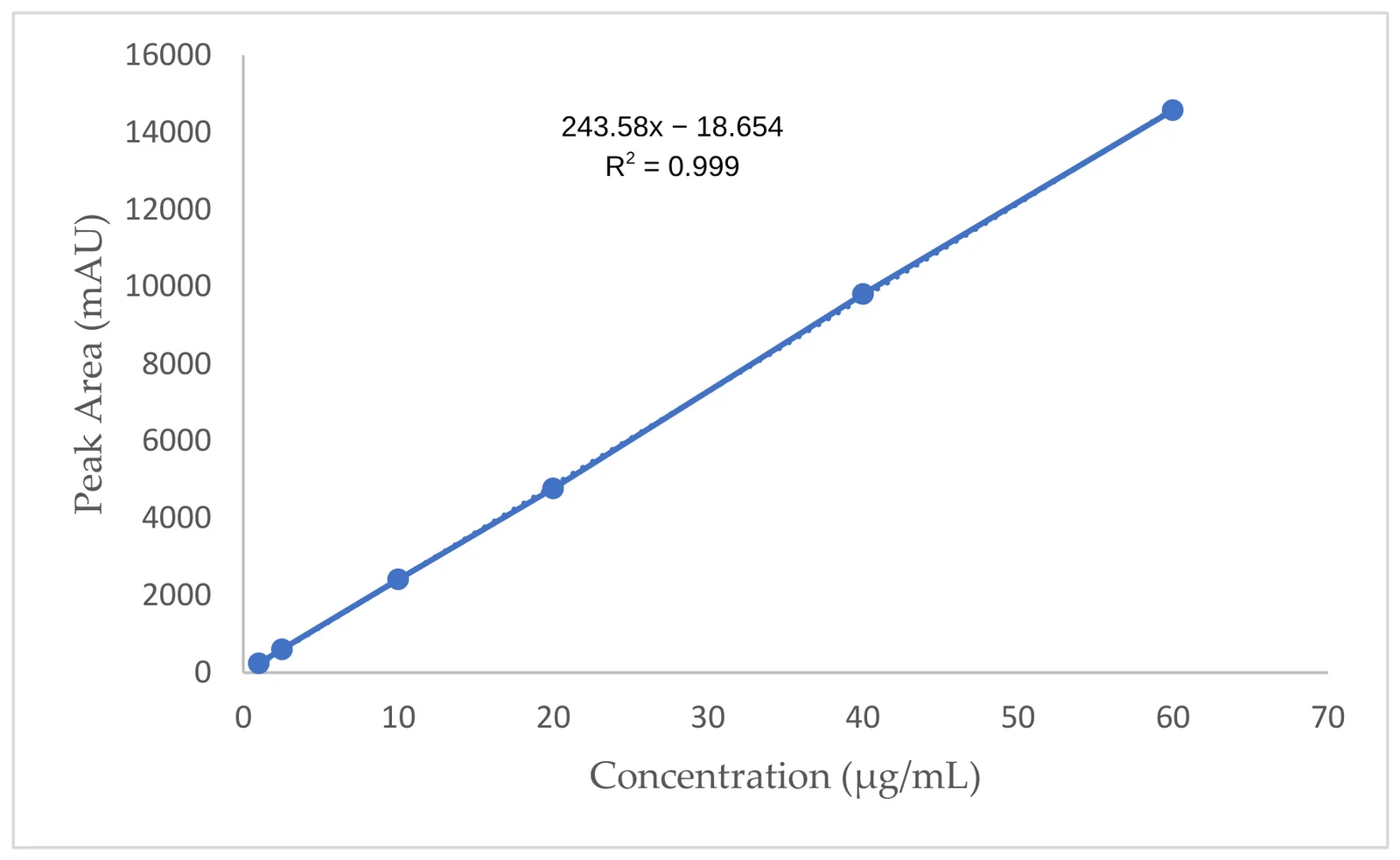
Calibration curve (plot of peak area versus concentration).

The linearity of an analytical method is its ability to produce results within a specific range that are directly proportional to the concentration of the analyte in the sample [36]. Linear regression analysis was performed for peak area versus concentration (Figure 3). The developed method exhibited linearity over the concentration range of 1–60 μg/mL. The calibration equation was as follows:

y = 243.58x − 18.65,

where *y* represents the peak area and *x* represents the RP concentration (μg/mL). The coefficient of determination (R^2^) was 0.9999, confirming excellent linearity of the calibration model and indicating that the proposed method provides highly reliable quantitative analysis within the evaluated concentration range.

#### 3.1.3. Accuracy

**Figure 4:**
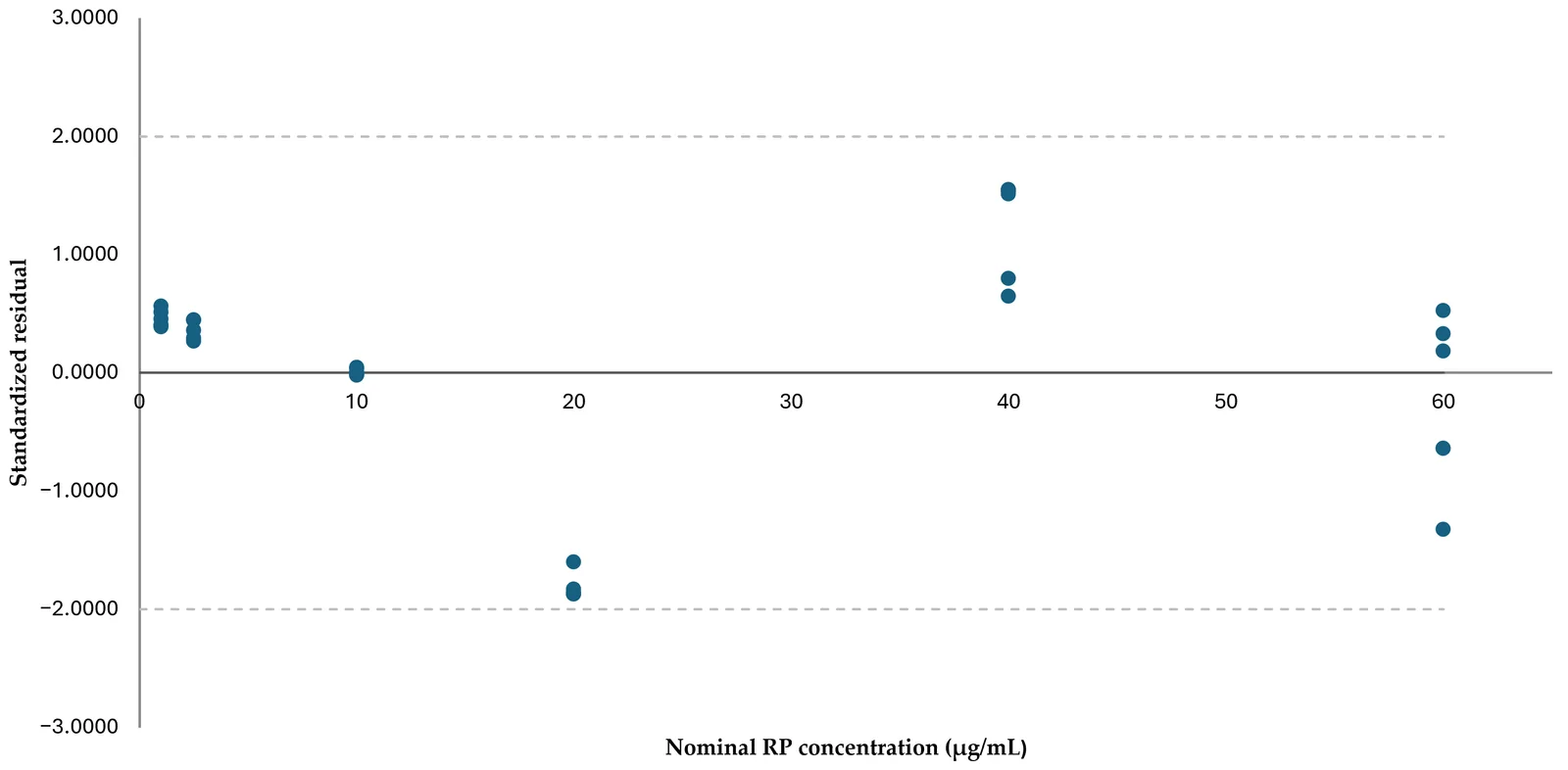
Plot of standardized residuals versus concentration. Blue dots represent individual standardized residuals for each experimental concentration run.

Percent recovery ranged from 99.77% to 104.80%, and RSD% remained below 2.0% at all concentration levels (Table 1). These results meet the acceptance criteria of the ICH guideline Q2(R2), confirming the accuracy of the method for determining RP. Residuals were calculated as the differences between the experimentally observed and regression-predicted peak areas and were plotted against RP concentration. The residuals were randomly distributed around zero (Figure 4), with no apparent systematic trend or concentration-dependent pattern, supporting the suitability of the linear regression model over the investigated range.

**Table 1:** Accuracy Results (mean ± SD; n = 3).

Theoretical Concentration (µg/mL)	Measured Concentration (μg/mL) ± SD	% Recovery	Precision (RSD%)
1	1.048	104.80	1.38
10	9.987	99.87	0.32
60	59.862	99.77	0.60

#### 3.1.4. Sensitivity

The mean signal-to-noise ratio obtained from the 1 µg/mL RP standard solution was 29.4. Based on this experimentally measured response, the LOD and LOQ were estimated to be 0.10 and 0.34 µg/mL, respectively. The estimated LOQ was lower than the lowest calibration standard; therefore, the validated quantitative range of the procedure was retained as 1–60 µg/mL.

#### 3.1.5. Precision

Testing at low, medium, and high concentrations (1, 10, and 60 μg/mL) shows that the proposed method maintains consistent precision throughout the linear range (Table 2). Low RSD% values (<2%) indicate minimal random error and high repeatability during routine analysis. These results confirm that concentration changes do not impact the repeatability or reliability of quantitative RP determination.

**Table 2:** Precision Results (mean ± SD; n = 3).

Nominal Concentration (µg/mL)	Intraday		Interday (Three Consecutive Days)	
	Measured Conc. (μg/mL) ± SD	Precision RSD%	Measured Conc. (μg/mL) ± SD	Precision RSD%
1	1.074 ± 0.005	0.50	1.078 ± 0.020	1.85
10	10.038 ± 0.004	0.04	9.995 ± 0.035	0.35
60	59.941 ± 0.077	0.13	59.898 ± 0.324	0.54

#### 3.1.6. Solution Stability

The RP concentration at the initial time point was 40.63 ±0.486 µg/mL. After storage at room temperature for 24 and 48 h, the measured concentrations were 40.51 ± 0.004 and 39.85 ± 0.029 µg/mL, corresponding to 40.63 ± 0.486% of the initial concentration, respectively (Table 3). The RSD values remained below 2% throughout the study. These results demonstrated that the RP analytical solution was stable at room temperature for at least 48 h under the investigated conditions.

**Table 3:** Solution Stability Results (mean ± SD; n = 3).

Time (h)	RP Concentration (µg/mL)	Change %	RSD%
0	40.63 ± 0.486	-	1.20
24	40.51 ± 0.004	0.30	0.01
48	39.85 ± 0.029	1.92	0.07

#### 3.1.7. Robustness

To evaluate the robustness of the developed method, small and deliberate variations were introduced in key chromatographic parameters, including column temperature (20, 25, and 30 °C), flow rate (0.81, 0.90, and 0.99 mL/min), and detection wavelength (253, 254, and 255 nm) (Table 4). The effects of these variations on retention time and peak width were assessed using percent relative standard deviation (RSD%) values. The retention time and peak width RSD% values for all examined parameters remained below the widely accepted 2% threshold for analytical method validity. These results indicate that the developed HPLC method remains robust to minor, intentional changes in chromatographic conditions. Consequently, the method can provide reliable and reproducible results across different laboratory settings and analysis periods.

**Table 4:** Robustness Results (mean ± SD; n = 3).

Chromatographic Conditions	Value	Peak Area	RSD%	Retention Time	RSD%	Peak Width	RSD%
Temperature	20	8870.2 ± 49.43	0.56	7.16 ± 0.04	0.49	0.35 ± 0.002	0.63
	25	8643 ± 57.17	0.66	6.78 ± 0.05	0.72	0.32 ± 0.003	0.88
	30	8797.86 ± 66.17	0.75	6.74 ± 0.13	1.87	0.33 ± 0.002	0.64
Flow Rate (mL/dk)	0.81	9640.7 ± 84.92	0.88	7.53 ± 0.02	0.25	0.36 ± 0.004	1.01
	0.90	8643 ± 57.17	0.66	6.78 ± 0.05	0.72	0.32 ± 0.003	0.88
	0.99	7907.6 ± 101.26	1.28	6.06 ± 0.02	0.27	0.30 ± 0.001	0.31
Wavelength (nm)	253	9332.5 ± 46.37	0.50	6.78 ± 0.05	0.72	0.32 ± 0.001	0.32
	254	8643 ± 57.17	0.66	6.78 ± 0.05	0.72	0.32 ± 0.003	0.88
	255	7868.2 ± 39.75	0.51	6.78 ± 0.05	0.72	0.41 ± 0.002	0.41

#### 3.1.8. Phase-Solubility Study Results

**Figure 5:**
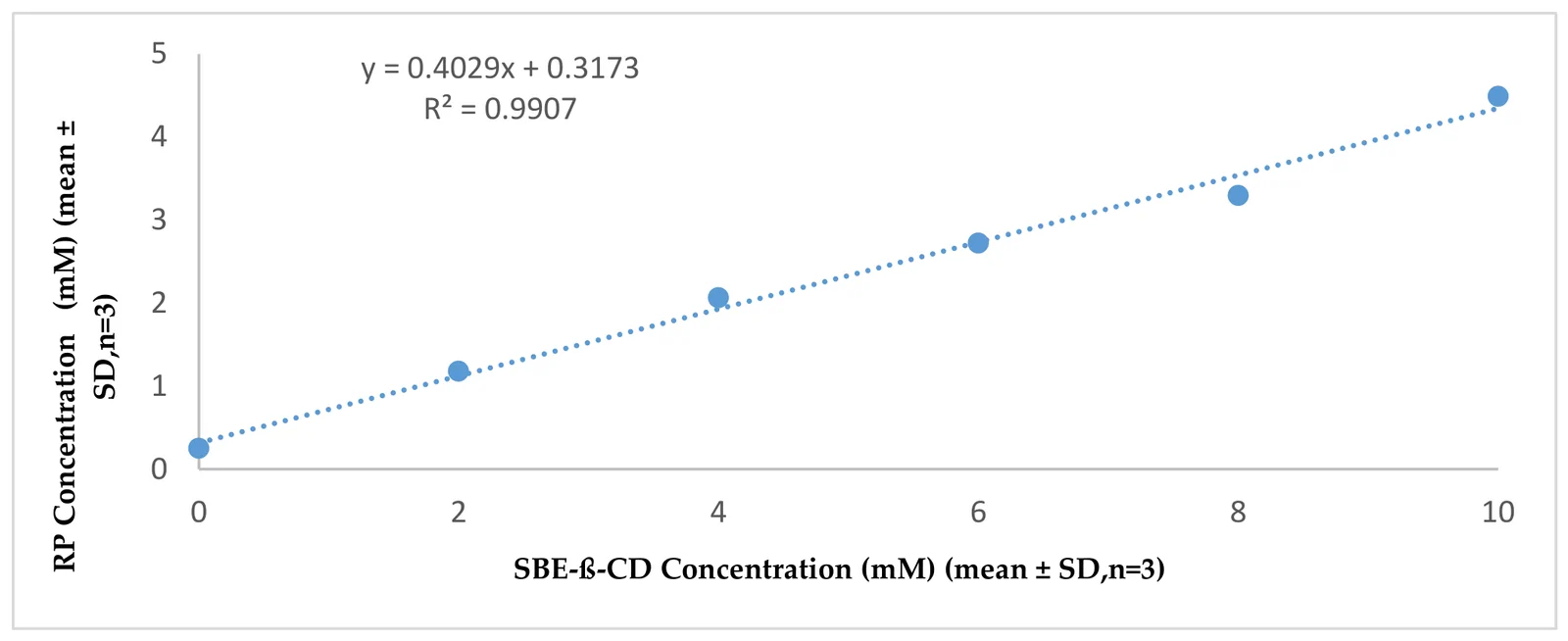
Phase solubility diagram of Reproxalap with SBE-β-CD in deionized water.

**Figure 6:**
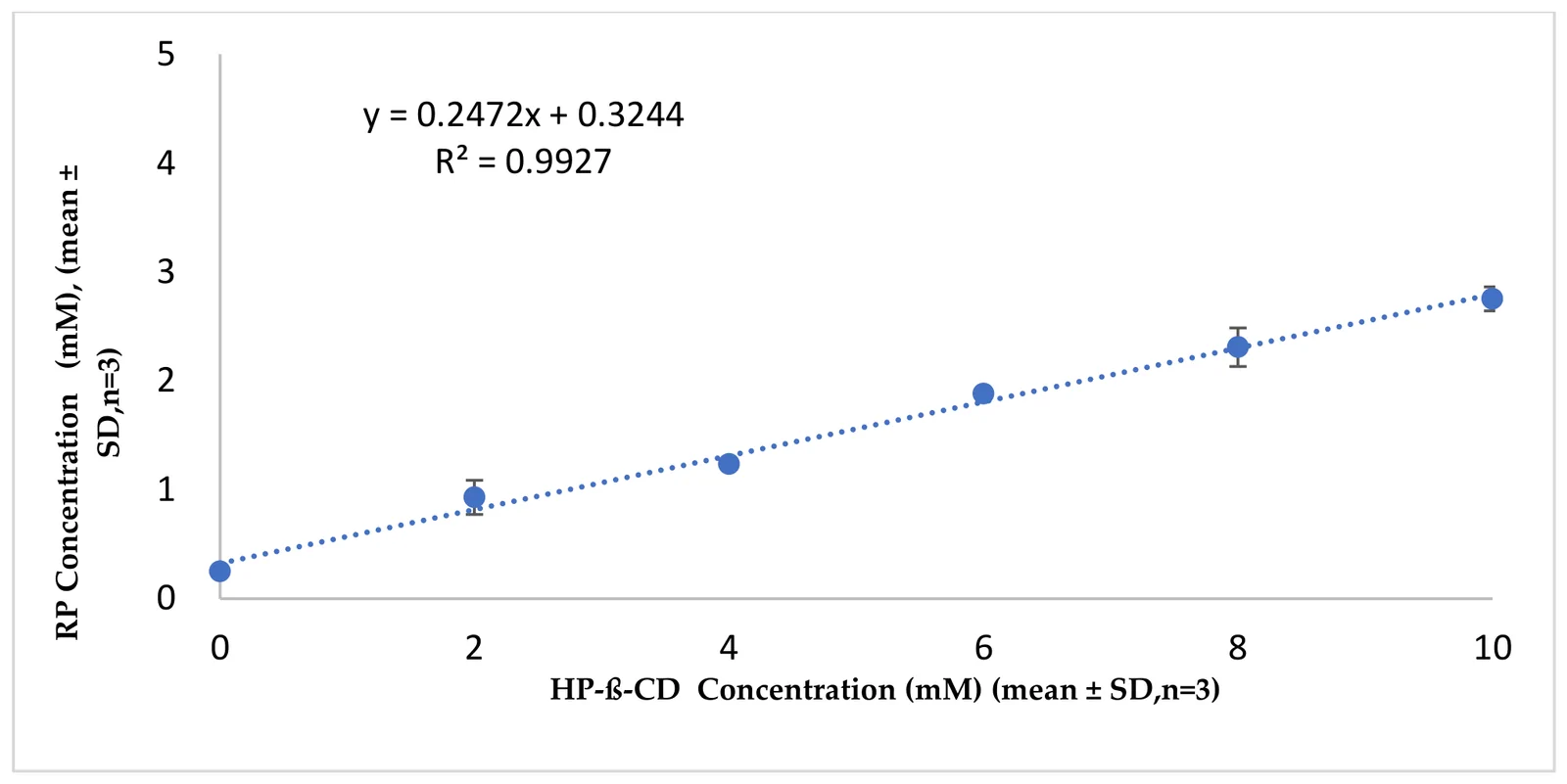
Phase solubility diagram of Reproxalap with HP-β-CDs in deionized water.

The stoichiometry and apparent stability constants of the RP: SBE-β-CD and RP: HP-β-CD inclusion complexes were evaluated using phase-solubility analysis. Phase-solubility diagrams were generated at 25 °C by plotting the apparent equilibrium concentration of RP against the concentrations of SBE-β-CD and HP-β-CD. As shown in Figure 5 and Figure 6, the apparent solubility of RP increased with increasing CD concentration over the concentration range of 0–10 mM for both derivatives. This increase in RP solubility suggests an interaction between RP and CDs. The resulting linear profiles correspond to an AL-type phase-solubility diagram, as described by Higuchi and Connors [37].

The slope (0.22 and 0.40 for SBE-β-CD and HP-β-CD, respectively) was <1, indicating a soluble inclusion complex with a 1:1 (guest:host) molar ratio. The stability constant (K1:1) reflects the binding strength between guest and host. Values between 100 and 5000 M^−1^ are generally considered optimal [38]. The apparent stability constant (K1:1), calculated from the slope of the phase-solubility plot, was 2666.66 and 526.32 M^−1^ and complex efficiency was 0.66 and 0.32 for SBE-β-CD and HP-β-CD, respectively—both within the range expected for a stable inclusion complex. For another hydrophobic drug, Semcheddine et al. reported K1:1 values of 1410.6 and 495.1 M^−1^ for SBE-β-CD and HP-β-CD, respectively. The agreement between these values and ours shows that our phase-solubility results are consistent with the literature, supporting the reliability of the data [8].

#### 3.1.9. Fourier Transform Infrared Assay

**Figure 7:**
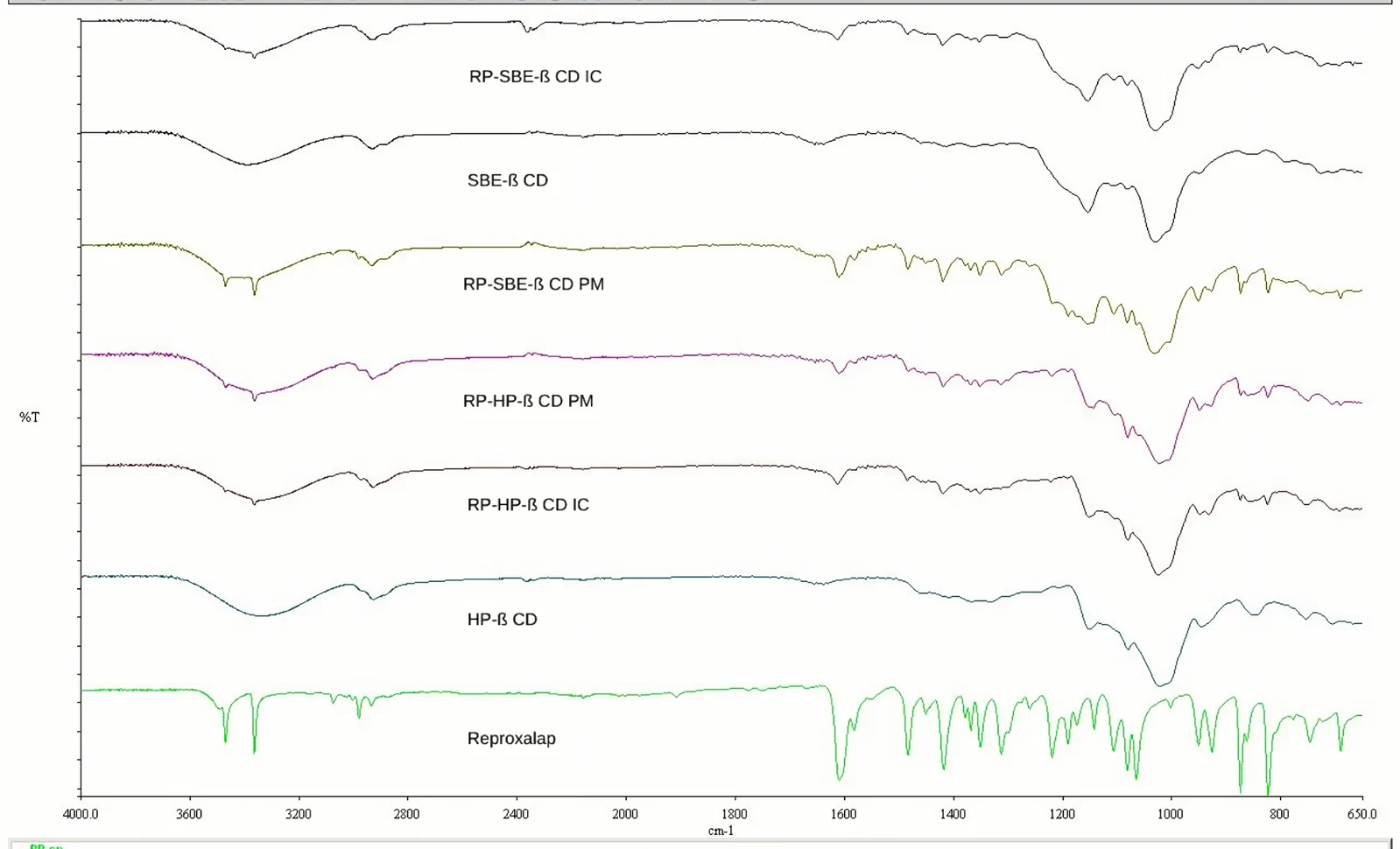
Results for FTIR spectra of pure SBE-β-C, pure HP-β-CD, RP: SBE-β-CD IC (RP: SBE-β-CD inclusion complex), RP: HP-β-CD IC (RP: HP-β-CD inclusion complex), RP: HP-β-CD PM (RP: HP-β-CD physical mixture), RP: SBE-β-CD PM (RP: SBE-β-CD physical mixture) and pure Reproxalap.

The FTIR spectrum (Figure 7) of RP shows characteristic bands consistent with its molecular structure. A doublet at approximately 3470 cm^−1^ and 3363 cm^−1^ corresponds to the asymmetric and symmetric stretching vibrations of the primary amine NH2 group, the molecule’s reactive center. Bands at 1609 and 1582 cm^−1^ correspond to the C=C and C=N stretching of the chlorinated quinoline ring. The aliphatic and aromatic C-H stretches (2980–3073 cm^−1^, coupled with the complex fingerprint region displaying significant C-Cl and C-N interactions between 1000 cm^−1^ and 1300 cm^−1^) further confirm the identity of RP.

The characteristic bands of RP disappeared in both inclusion complexes (RP: SBE-β-CD IC and RP: HP-β-CD IC). In the physical mixtures RP: SBE-β-CD physical mixture (RP: SBE-β-CD PM) and RP: HP-β-CD physical mixture (RP: HP-β-CD PM), however, the RP bands were more intense than in the complexes. The complex spectra more closely resembled those of the pure cyclodextrins. As in previous reports, the loss of the characteristic RP bands suggests that the drug was successfully incorporated into the cyclodextrin cavities [39,40].

#### 3.1.10. Differential Scanning Calorimetry

Further confirmation of inclusion complex formation was obtained by DSC. It has been reported that the melting and sublimation transitions of guest molecules may shift or disappear when they are incorporated into crystal lattices or CD cavities [41].

**Figure 8:**
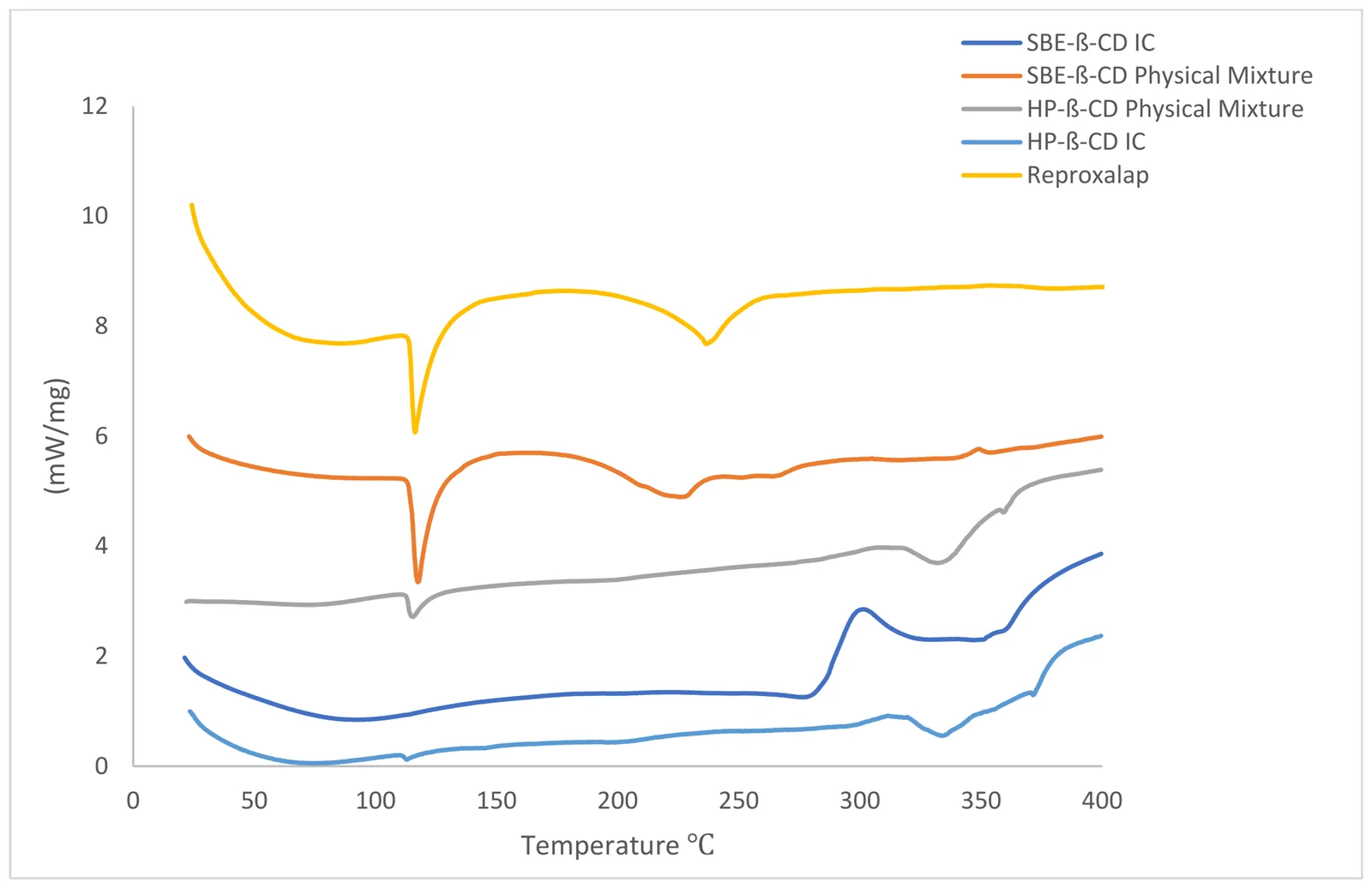
Differential scanning calorimetry thermograms of RP: SBE-β-CD IC (RP: SBE-β-CD inclusion complex), RP: HP-β-CD IC (RP: HP-β-CD inclusion complex), RP: HP-β-CD PM (RP: HP-β-CD physical mixture), RP: SBE-β-CD PM (RP: SBE-β-CD physical mixture) and pure Reproxalap.

The DSC thermograms (Figure 8) showed that the characteristic melting peak of RP at 116.0 °C was partially retained in the physical mixtures with RP: SBE-β-CD PM and RP: HP-β-CD PM, indicating that RP largely remained in its crystalline form in these systems. In contrast, the disappearance of this peak in the inclusion complexes suggests a substantial reduction in RP crystallinity following complexation, likely because RP is encapsulated within the hydrophobic cavities of the CDs. These findings are consistent with previous reports indicating that the disappearance or reduction in the thermal transition of a guest molecule is a key indicator of inclusion complex formation with CDs.

#### 3.1.11. Solubility Study

CDs form reversible inclusion complexes by encapsulating poorly water-soluble drug molecules into their hydrophobic cavities via noncovalent interactions, thereby enhancing drug solubility [42]. RP exhibits low solubility in deionized water. In this study, its solubility was improved by forming CD inclusion complexes, consistent with previous reports involving other poorly water-soluble drugs [43].

The solubility of RP in deionized water (0.25 mM) increased 8.6-fold to 2.15 ± 0.43 mM upon complexation with HP-β-CD. In contrast, the RP: SBE-β-CD inclusion complex produced an 22.4-fold increase in solubility, yielding 5.6 ± 0.98 mM. These findings are consistent with previous reports; for example, the aqueous solubility of simvastatin, a poorly water-soluble drug, was enhanced through complexation with HP-β-CD [44]. However, RP exhibited greater solubility enhancement with SBE-β-CD than with HP-β-CD.

This difference is attributed to the four-carbon butyl chain and to the electrostatic repulsion between negatively charged terminal groups of SBE-β-CD, which extends the hydrophobic cavity and increases its affinity for drug molecules [45]. Similarly, Kulkarni and Belgamwar reported a marked increase in the aqueous solubility of the poorly water-soluble anticancer agent chrysin (CHR) following complexation with SBE-β-CD, increasing from 0.035 mg/mL (free CHR) to 3.80 mg/mL [46].

#### 3.1.12. Moisture Content and Yield

The moisture content of the inclusion complexes was 2.2 ± 0.03% for RP: SBE-β-CD IC and 2.9 ± 0.05% for RP: HP-β-CD IC, and the yields were 82 ± 1.83% for RP: SBE-β-CD IC and 79 ± 1.32% for RP: HP-β-CD IC (n = 3).

#### 3.1.13. Characterization of In Situ Gel Formulation

**Figure 9:**
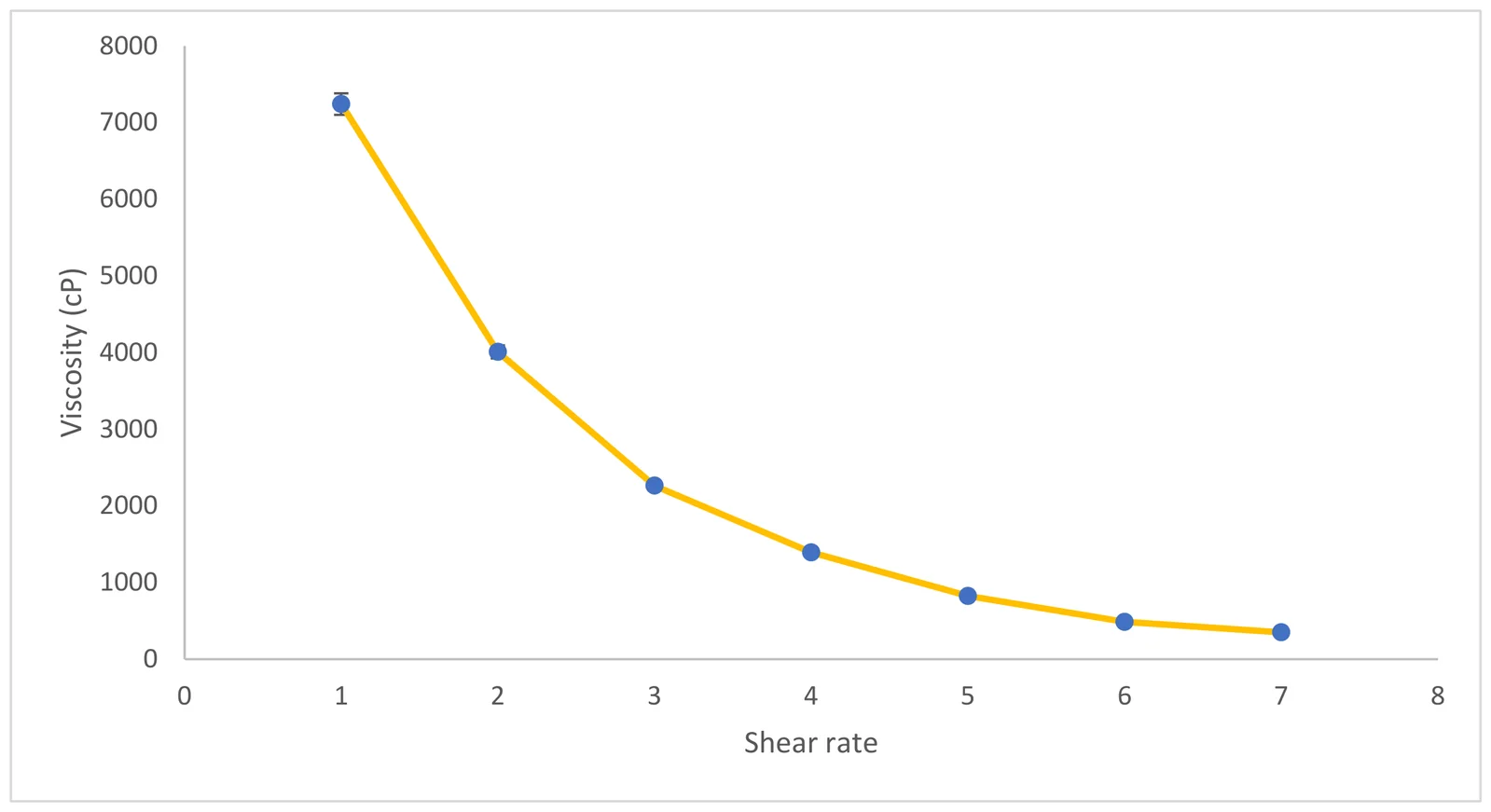
Rheological behavior of hydrogel (n = 3).

The pH of the hydrogel was found to be around 7.5. A literature review indicates that ophthalmic applications are suitable within a pH range of 4 to 8 [47]. Therefore, considering these findings, it can be concluded that each formulation is suitable for ocular application. The prepared hydrogels exhibited transparency, which is not expected to pose a problem for patient compliance during application. Thermosensitive gels are typically liquids at room temperature and convert into a gel at body temperature. The polymers most often used to make these systems are Poloxamers [48]. Among the Poloxamer family of non-ionic surfactant poloxamers, Pluronic 407 is widely used in ophthalmic formulations because it is clear, colorless, and easy to handle. In ocular delivery, thermosensitive gels are attractive because they can be applied as liquids at about 25 °C and then gel when they contact the eye surface, where the temperature is around 32 °C. This temperature-triggered gelation helps the formulation stay on the eye longer and improves ocular bioavailability [18]. Viscosity values are given in Table 5. It was observed that the hydrogels showed a fourfold increase in viscosity between 27 °C and 29 °C, indicating the onset of gelation. Subsequently, at 33 °C, gelation could be completed on the ocular surface, with a viscosity between 90.000–95,000 cP. To evaluate rheological behavior, viscosity measurements performed at varying rotational speeds revealed a progressive decrease in the viscosity of the hydrogel formulation with increasing rpm, indicating pseudoplastic (shear-thinning) flow behavior (Figure 9). This finding is consistent with previous reports describing similar rheological characteristics for in situ gel systems in the literature [33].

### 3.2. Application of the RP-HPLC Method for Determination of the Release Profile of the Hydrogel Formulation

**Figure 10:**
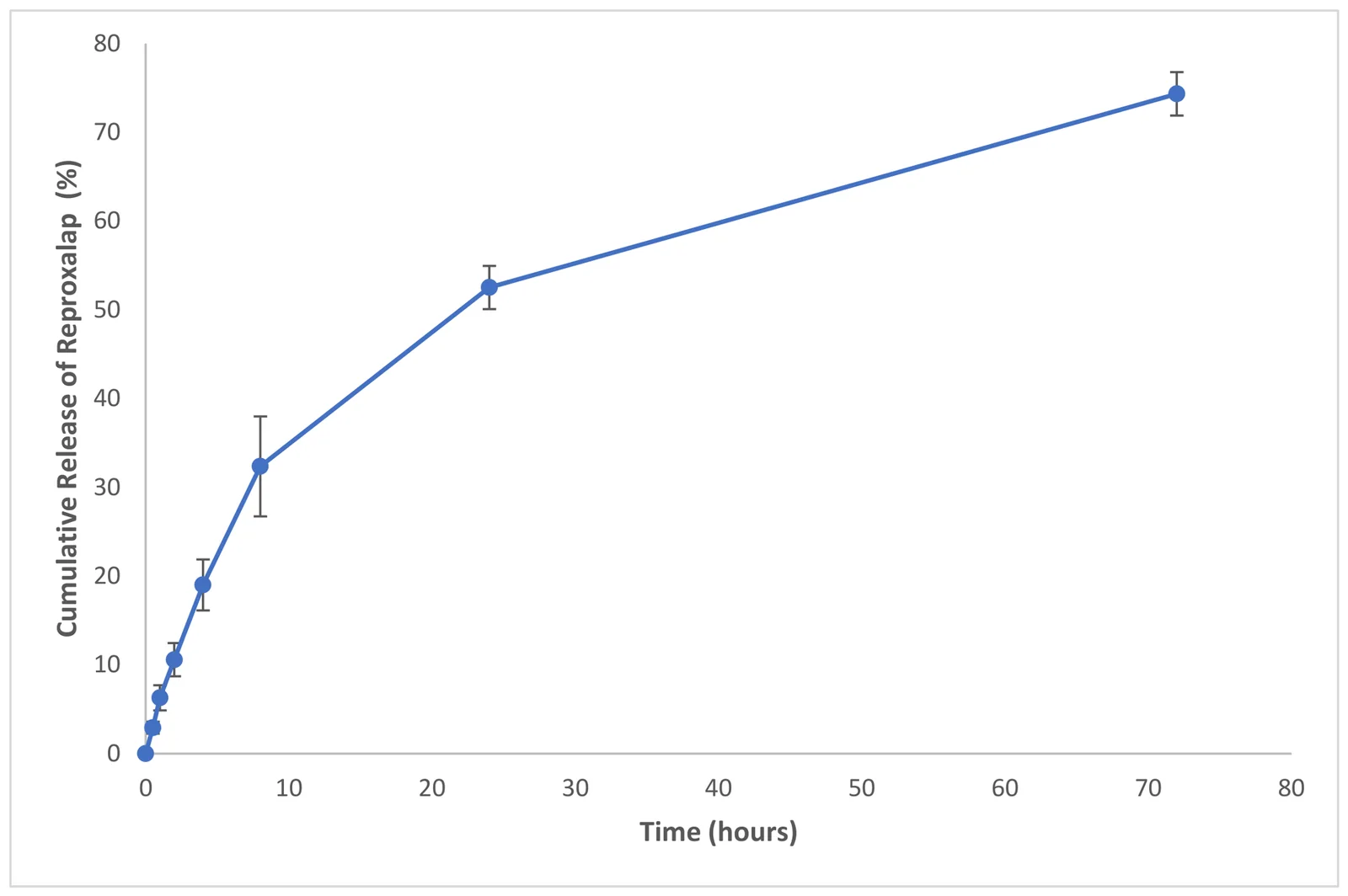
Percent Cumulative Release of RP from Hydrogel Formulation Over Time.

The release profile of the hydrogel formulation is presented in Figure 10. RP release reached 50% within 24 h and 70% after 72 h. The developed RP-HPLC method enabled the selective analysis of RP without interference from the CD derivatives present in the hydrogel matrix. To the best of our knowledge, no HPLC method has previously been reported for the analysis of RP. The developed method has a run time of less than 10 min, which may facilitate its application in future formulation and analytical studies.

Mathematical modeling facilitates the quantitative analysis of release kinetics, which is essential for determining the fate of drug molecules released from carrier platforms. Release kinetic studies enable the calculation of parameters such as AIC, R^2^, and other constants that define drug release mechanisms. Through mathematical models, properties of drug carrier platforms, including drug release behavior, hydration, swelling, and erosion of matrix tablets, can be elucidated. Consequently, various mathematical formulae were employed to clarify the release kinetic model of the formulation. Four different models were scrutinized based on R^2^, R^2^adjusted, AIC and MSC. In Table 6, among the tested models, the Higuchi model provided the best mathematical fit to the dissolution data (R^2^ = 0.973, lowest AIC, highest MSC), indicating that the overall release kinetics can be adequately described by this model. However, the Korsmeyer–Peppas model was used to further elucidate this mechanism. The release exponent (n) obtained from the Korsmeyer–Peppas model was 0.65, which falls within the range characteristic of anomalous (non-Fickian) transport, suggesting that drug release is driven by a combination of diffusion and polymer chain relaxation/erosion rather than pure Fickian diffusion. These results align with existing literature in P407/HA systems. Nguyen et al. [49] similarly reported that the Higuchi model provided the optimal fit for P407/HA gel formulations (R^2^ = 0.99), suggesting a combination of Fickian diffusion and matrix relaxation with the Korsmeyer–Peppas model n value.

**Table 6:** Release kinetic models and results of hydrogel (mean ± SD; n = 3).

	Model and Equation/Formulation			
Evaluation Criteria	Zero-Order F = k0×t	Higuchi F = kH×t^0.5	Hixson–Crowell F = 100 · [1 − (1 − kHC·t)^3^]	Korsmeyer–Peppas F = kKP×t^n
R^2^	0.692	0.973	0.857	0.834
Adjusted R^2^	0.692	0.973	0.857	0.806
AIC	60.638	41.240	54.516	57.705
MSC	0.795	3.220	1.561	1.162
n	-	-	-	0.65

## 4. Conclusions

RP is a novel anti-inflammatory molecule and RASP inhibitor under development for the treatment of DED. To date, no validated HPLC method has been reported for quantifying RP. In this study, an HPLC method was developed and validated in accordance with the ICH guideline Q2(R2). The method enabled accurate and precise quantification of RP, with an LOQ of 0.34 µg/mL.

In this study a thermosensitive in situ gel platform composed of P407 and hyaluronic acid-based in situ gel, frequently preferred for ophthalmic drug delivery systems in the literature, was designed, and an RP: SBE-β-CD inclusion complex was successfully loaded into this gel matrix. Phase-solubility studies demonstrated that RP could form stable 1:1 molar inclusion complexes with both HP-β-CD and SBE-β-CD. FTIR and DSC analyses showed consistent evidence of inclusion complex formation, as reflected by the reduction or disappearance of characteristic RP signals and thermal transitions. Compared with the HP-β-CD inclusion complex, the RP: SBE-β-CD inclusion complex exhibited superior performance, as evidenced by a higher stability constant and a greater increase in RP solubility in the phase-solubility studies. The developed hydrogel remained in the sol state at room temperature and underwent a sol–gel transition at ocular surface temperature (≈29–33 °C), indicating that it can be easily instilled as a liquid at room temperature and converted into a gel upon contact with the ocular surface. The in vitro release data best fit the Higuchi model (R^2^ = 0.973), and the Korsmeyer–Peppas model release exponent value (n = 0.65) falls within the anomalous (non-Fickian) transport range (0.5 < n < 1), suggesting that drug release from the hydrogel formulation is driven by a combination of diffusion and polymer chain relaxation/erosion.

The RP-HPLC method developed in this study demonstrated that RP could be determined selectively and reproducibly within this complex gel matrix, without any interference. The specificity of the method showed that chromatographic separation was preserved even in the complex matrix environment jointly formed by poloxamer, hyaluronic acid, and cyclodextrin; the fact that all parameters—linearity, accuracy, precision, LOD/LOQ, robustness, and system suitability—remained within the acceptance criteria confirmed that the method is a reliable analytical tool for this type of formulation. Deliberate small changes in chromatographic conditions did not significantly affect the response, demonstrating that the method’s robustness holds not only theoretically but also under practical formulation conditions. Therefore, the main contribution of this study is demonstrating that RP–cyclodextrin inclusion complexes loaded into poloxamer/hyaluronic acid-based in situ gels can be successfully analyzed using an RP-HPLC method validated in accordance with the ICH Q2(R2) guideline; this indicates that the method can serve as a reference for future development and quality control studies of RP in drug delivery systems.

## Figures and Tables

**Table 5:** Viscosity changes against temperature of hydrogel formulations (n = 3).

Temperature (°C)	25	27	29	33
Viscosity value (cP)	2.000–2.500	10.000–12.500	40.000–45.000	90.000–95.000

## Data Availability

The original contributions presented in this study are included in the article. Further inquiries can be directed to the corresponding author.

## Abbreviations

DED	Dry Eye Disease
RASP	Reactive Aldehyde Species
RP	Reproxalap
CD	Cyclodextrin
α-CD	Alpha-Cyclodextrin
β-CD	Beta-Cyclodextrin
γ-CD	Gamma-Cyclodextrin
HP-β-CD	Hydroxypropyl-β-Cyclodextrin
SBE-β-CD	Sulfobutyl Ether β-Cyclodextrin
RP: HP-β-CD IC	Reproxalap Hydroxypropyl Ether β-Cyclodextrin Inclusion Complex
RP: SBE-β-CD IC	Reproxalap Sulfobutyl Ether β-Cyclodextrin Inclusion Complex
RP: SBE-β-CD PM	Reproxalap Sulfobutyl Ether β-Cyclodextrin Physical Mixture
RP: HP-β-CD PM	Reproxalap Hydroxypropyl Ether β-Cyclodextrin Physical Mixture
P407	Poloxamer 407
HPLC	High-Performance Liquid Chromatography
RP-HPLC	Reverse-Phase High-Performance Liquid Chromatography
ICH	International Council for Harmonisation
IUPAC	International Union of Pure and Applied Chemistry
